# Supplementary material for: Flexible Modeling of Net Survival and Cure by AML Subtype and Age: A French Population-Based Study from FRANCIM
Source: J Clin Med. 2021 Apr 13;10(8):1657. doi: 10.3390/jcm10081657 (PMC8069423; doi:10.3390/jcm10081657)

SUPPLEMENTARY METHODS

Model building strategy in the modelisation of effects of time since diagnosis and age on the excess mortality rate and on statistical cure.

We explain here the strategy used to model excess mortality and the effect of covariates (time since diagnosis, age) on : i) excess mortality using the flexible model proposed by Nelson *et al.*[13] and ii) statistical cure using the flexible cure model proposed by Andersson *et al*[14]. These two models are fitted to the logarithm of the cumulative excess mortality using restricted cubic spline function and based on continuous modelling of the baseline hazard. Compared to the model proposed by Nelson *et al.*, the flexible cure model proposed by Andersson *et al.*: i) added a condition to force the cumulative excess mortality rate to be constant after the last knot of time since diagnosis; and ii) add a supplemental knot to model the Non-Linear effect of the baseline hazard at the 99th percentiles of the observed death times. Estimations from the model with the cure assumption and without cure assumption were graphically compared to assess adequacy of net survival curves with the non-parametric estimator proposed by Pohar-Perme and to assess adequacy of excess mortality rate with those obtained using a model based on a step function to model baseline hazard (**Supplementary Figure 1-11**).

One model was performed independently for each AML-subtype in both sex. Time since diagnosis and age are considered as continuous function. The model assuming a time-dependent and non-linear effect of age was privileged when the number of cases was sufficient. When it was not the case, the model was based on a non-linear and a proportional-hazard effect of age.
In situation where conditions fixed was accepted (number of cases at diagnosis higher than 500 or numbers of deaths at 10-years after AML diagnosis higher than 130), functions used in the modelisation were defined here:

Effect	Function	Kind of function	Location of the knot	
			Flexible excess mortality model	Flexible cure model
**Baseline hazard**	f	Restrictive Cubic spline	According to AML subtype and Sex	According to AML subtype and Sex
**Linearity of age**	g	Quadratic spline	At mean age	At mean age
**Time-Dependent effect of age**	h	Restrictive Cubic spline	At the 1st, 25th, 50th, 75th, 95th percentiles and 12 years after the diagnosis	Supplemental knot at 99th percentile

If the number of cases at diagnosis less than 500 and numbers of death at 10-years after AML diagnosis was less than 130, we performed new strategy reducing the number of model parameters as regards to the number of events at 10-years. So, we eliminate a parameter until to respecting the criteria of “one parameter per 10 deaths” in order as follow: 1) drop the 95th percentiles to model baseline hazard (i.e. 11

parameters in the model); 2) drop the 75th percentiles to model baseline hazard (i.e 9 parameters in the model); 3) to model age with a non-linear and proportional effect (i.e. 6 parameters in the model)

AML-subtypes with less than 70 deaths until 10 years were not retained in survival analysis.

The model retained for each AML-subtype and sex (and flexible functions used) are presented in the following table with on the left column, final model retained using the flexible excess mortality model and on the right column, model retained using the flexible cure model.

	Flexible excess mortality model (Nelson's model)		Flexible cure model (Andersson's model)	
**MEN**	**Final model retained**	**Baseline hazard**	**Final model retained**	**Baseline hazard Supplémental knots**
**Acute promyelocytic leukemia (APL)**	f(Time) + g(Age) + h(Time) x Age	at 1st, 25th, 50th, 75^{th,} percentiles and 12 years	f(Time) + g(Age) + h(Time) x Age	at 99th percentile
**AML with recurrent cytogenetic abnormalities (AML-RCA)**	f(Time) + g(Age) + h(Time) x Age	at 1st, 25th, 50th percentiles and 12 years	f(Time) + g(Age) + h(Time) x Age	at 99th percentile
**AML with myelodysplasia-related changes (AML-MRC)**	f(Time) + g(Age)	at 1st, 25th, 50th, 75^{th,} 95th percentiles and 12 years	f(Time) + g(Age)	at 99th percentile
**Therapy-related AML; NOS (t-AML)**	f(Time) + g(Age) + h(Time) x Age	at 1st, 50th percentiles and 12 years	f(Time) + g(Age) + h(Time) x Age	at 25th, 75th, 95th, 99th percentile
**Pure erythroid leukemia (AML-M6)**	f(Time) + g(Age) + h(Time) x Age	at 1st, 50th, 95th percentiles and 12 years	f(Time) + g(Age) + h(Time) x Age	at 25th, 75th, 99th percentile
**Acute Myelomonocytic leukemia (AML-M4)**	f(Time) + g(Age) + h(Time) x Age	at 1st, 25th, 50th, 75^{th,} 95th percentiles and 12 years	f(Time) + g(Age) + h(Time) x Age	at 99th percentile
**Acute myeloid leukemia with minimal differentiation (AML-M0)**	f(Time) + g(Age) + h(Time) x Age	at 1st, 25th, 50th, 75^{th,} 95th percentiles and 12 years	f(Time) + g(Age) + h(Time) x Age	at 99th percentile
**Acute Myeloid leukemia without maturation (AML-M1)**	f(Time) + g(Age) + h(Time) x Age	at 1st, 25th, 50th, 75^{th,} 95th percentiles and 12 years	f(Time) + g(Age) + h(Time) x Age	at 99th percentile
**Acute Myeloid leukemia with maturation (AML-M2)**	f(Time) + g(Age) + h(Time) x Age	at 1st, 25th, 50th, 75^{th,} 95th percentiles and 12 years	f(Time) + g(Age) + h(Time) x Age	at 99th percentile
**Acute monoblastic and monocytic leuaemia (AML-M5)**	f(Time) + g(Age) + h(Time) x Age	at 1st, 25th, 50th, 75^{th,} 95th percentiles and 12 year	f(Time) + g(Age) + h(Time) x Age	at 99th percentile
**Acute Myeloid leukemia, not otherwise specified (AML-NOS)**	f(Time) + g(Age) + h(Time) x Age	at 1st, 25th, 50th, 75^{th,} 95th percentiles and 12 years	f(Time) + g(Age) + h(Time) x Age	at 99th percentile
**WOMEN**				
**Acute promyelocytic leukemia (APL)**	f(Time) + g(Age)	at 1st, 25th, 50^{th,} percentiles and 12 years	f(Time) + g(Age)	at 99th percentile
**AML with recurrent cytogenetic abnormalities (AML-RCA)**	f(Time) + g(Age) + h(Time) x Age	at 1st, 25th, 50th percentiles and 12 years	f(Time) + g(Age) + h(Time) x Age	at 99th percentile
**AML with myelodysplasia-related changes (AML-MRC)**	f(Time) + g(Age)	at 1st, 25th, 50th, 75^{th,} 95th percentiles and 12 years	f(Time) + g(Age)	at 99th percentile
**Therapy-related AML; NOS (t-AML)**	f(Time) + g(Age)	at 1st, 25th, 50th, 75^{th,} 95th percentiles and 12 years	f(Time) + g(Age)	at 99th percentile
**Pure erythroid leukemia (AML-M6)**	f(Time) + g(Age) + h(Time) x Age	at 1st, 50th, 95th percentiles and 12 years	f(Time) + g(Age) + h(Time) x Age	at 99th percentile
**Acute Myelomonocytic leukemia (AML-M4)**	f(Time) + g(Age) + h(Time) x Age	at 1st, 25th, 50th, 75^{th,} 95th percentiles and 12 years	f(Time) + g(Age) + h(Time) x Age	at 99th percentile
**Acute myeloid leukemia with minimal differentiation (AML_M0)**	f(Time) + g(Age)	at 1st, 25th, 50th, 75^{th,} 95th percentiles and 12 years	f(Time) + g(Age)	at 99th percentile
**Acute Myeloid leukemia without maturation (AML-M1)**	f(Time) + g(Age) + h(Time) x Age	at 1st, 25th, 50th, 75^{th,} 95th percentiles and 12 years	f(Time) + g(Age) + h(Time) x Age	at 99th percentile
**Acute Myeloid leukemia with maturation (AML_M2)**	f(Time) + g(Age) + h(Time) x Age	at 1st, 50th percentiles and 12 years	f(Time) + g(Age) + h(Time) x Age	at 25th, 75th, 95th, 99th percentile
**Acute monoblastic and monocytic leukemia (AML-M5)**	f(Time) + g(Age) + h(Time) x Age	at 1st, 50th percentiles and 12 years	f(Time) + g(Age) + h(Time) x Age	at 99th percentile
**Acute Myeloid leukemia, not otherwise specified (AML-NOS)**	f(Time) + g(Age) + h(Time) x Age	at 1st, 25th, 50th, 75^{th,} 95th percentiles and 12 years	f(Time) + g(Age) + h(Time) x Age	at 99th percentile

In the following, LL stands for “Log-Linear”, NL for “Non-Linear”, TD for “Time-Dependent”, PH for “Proportional-Hazard”.
Even if the model on which estimates are based mainly correspond to the full model adjusted on the age at diagnosis with NL and TD effect, we explored all the same the shape of effects of age using a model-building strategy on inference about non-linear (i.e. risk not constant between tow modality of a covariable) and time-dependent effect (i.e. risk not constant over time since diagnosis).

In each AML subtype and sex, we used a likelihood ratio test to study:
1) the non-linear effect of age assuming firstly a TD effect of age and secondly a PH effect of age,
2) the time-dependent effect of age assuming firstly a NL effect of age and secondly a LL effect of age.
The effect was considered statistically significant with a cut off equal to 0.05.
Results of likelihood ratio test by AML subtype and sex, are presented in the following table with on the left column, effect of age retained using the flexible excess mortality model and on the right column, effect of age retained using the flexible cure model. Functions used in the modelisation are the same that those presented previously.

P-value	Flexible excess mortality model (Nelson’s model)				Flexible cure model (Andersson’s model)			
	Linear effect		Time dependent effect		Linear effect		Time dependent effect	
	NL assuming TD	NL assuming PH	TD assuming NL	TD assuming LL	NL assuming TD	NL assuming PH	TD assuming NL	TD assuming LL
**MEN**								
**Acute promyelocytic leukemia with t(15;17)(q22;q12) (APL)**	0.017	0.024	0.014	0.019	0.013	0.021	0.002	0.004
**AML with recurrent cytogenetic abnormalities (AML-RCA)**	*0.106*	0.015	*0.0676*	0.011	0.018	0.011	*0.208*	*0.122*
**AML with myelodysplasia-related changes (AML-MRC)**	*not tested*	*0.243*	*not tested*	*not tested*	*not tested*	*0.232*	*not tested*	*not tested*
**Therapy-related AML; NOS (t-AML)**	*0.500*	*0.355*	0.042	0.031	*0.386*	*0.305*	*0.155*	*0.123*
**Pure erythroid leukemia (AML-M6)**	*0.256*	*0.415*	*0.148*	*0.222*	*0.308*	*0.327*	*0.426*	*0.451*
**Acute Myelomonocytic leukemia (AML-M4)**	0.009	0.001	*0.339*	*0.070*	0.008	0.002	*0.185*	0.037
**Acute myeloid leukemia with minimal differentiation (AML-M0)**	*0.794*	*0.275*	0.003	0.001	*0.728*	*0.259*	0.001	< 0.001
**Acute Myeloid leukemia without maturation (AML-M1)**	0.001	0.001	*0.088*	*0.079*	0.002	0.001	*0.094*	0.031
**Acute Myeloid leukemia with maturation (AML-M2)**	0.019	0.025	0.001	0.001	0.031	0.017	0.001	0.001
**Acute monoblastic and monocytic leukemia (AML-M5)**	0.048	0.003	0.028	0.002	0.013	0.003	*0.127*	0.027
**Acute Myeloid leukemia, not otherwise specified (AML-NOS)**	0.002	< 0,001	*0.092*	0.025	0.002	< 0.001	*0.068*	0.008
**WOMEN**								
**Acute promyelocytic leukemia with t(15;17)(q22;q12) (APL)**	*not tested*	*0.104*	*not tested*	*not tested*	*not tested*	*0.066*	*not tested*	*not tested*
**AML with recurrent cytogenetic abnormalities (AML-RCA)**	0.044	0.025	*0.998*	*0.556*	0.024	0.024	*0.696*	*0.734*
**AML with myelodysplasia-related changes (AML-MRC)**	*not tested*	*0.944*	*not tested*	*not tested*	*not tested*	*0.946*	*not tested*	*not tested*
**Therapy-related AML; NOS (t-AML)**	*not tested*	*0.147*	*not tested*	*not tested*	*not tested*	*0.149*	*not tested*	*not tested*
**Pure erythroid leukemia (AML-M6)**	*0.926*	*0.992*	*0.706*	*0.739*	*0.985*	*0.995*	*0.777*	*0.785*
**Acute Myelomonocytic leukemia (AML-M4)**	0.008	0.002	*0.099*	0.027	0.011	0.002	0.044	0.009
**Acute myeloid leukemia with minimal differentiation (AML-M0)**	*not tested*	*0.388*	*not tested*	*not tested*	*not tested*	*0.378*	*not tested*	*not tested*
**Acute Myeloid leukemia without maturation (AML-M1)**	< 0,001	0.003	0.034	< 0,001	< 0,001	0.002	0.028	0.001
**Acute Myeloid leukemia with maturation (AML-M2)**	0.008	0.016	*0.055*	*0.103*	0.028	0.007	*0.151*	0.039
**Acute monoblastic and monocytic leukemia (AML-M5)**	0.001	< 0,001	*0.828*	*0.524*	< 0,001	< 0,001	*0.309*	*0.654*
**Acute Myeloid leukemia, not otherwise specified (AML-NOS)**	*0.317*	*0.092*	*0.096*	0.032	*0.272*	*0.086*	0.037	0.012

Supplementary Figure 1-11:

Adequacy of flexible excess model and flexible cure model in each Acute Myeloid Leukemia subtype by sex: A) in Men and B) in Women using:

1) Estimation of probability of net survival compared with those obtained using the non-parametric estimator of Pohar-Perme to estimate:
   - Probability of net survival (%) over time since diagnosis for all age (Top left graph),
   - One-year net survival and CI95% over age at diagnosis in years (Top middle graph),
   - Five-year net survival and CI95% over age at diagnosis in years (Top right graph).

   Note that for Pohar-Perme estimator, net survival was estimated separately per age-group in 10 year intervals;
2) Estimation of excess mortality rate compared with those obtained using a model with a step function to model baseline hazard for estimation of excess mortality rate over time from diagnosis in three age-groups:
   - for patient of [15-44] years old (Bottom left graph),
   - for patient of [45-64] years old (Bottom middle graph),
   - for patient equal or more than 65 years old (Bottom right graph).

   The flexible excess and cure models were adjusted, respectively, on the median age of each age-group and excess mortality was model using step function for baseline hazard separately in each age-group without other covariates.

Supplementary Figure 1 - Adequacy of flexible excess model and flexible cure model in acute promyelocytic leukemia (APL): A) Men and B) Women. Net survival (%) over time since diagnosis for all age (Top left graph). Net survival (and CI95%) over age at diagnosis in years at 1-year (Top middle graph) and 5-year (Top right graph). Excess mortality rate in deaths per person-year over time since diagnosis in years in [15-44] age-group (Bottom left graph), in [45-64] age-group (Bottom middle graph) and in 65 years old and more (Bottom right graph).

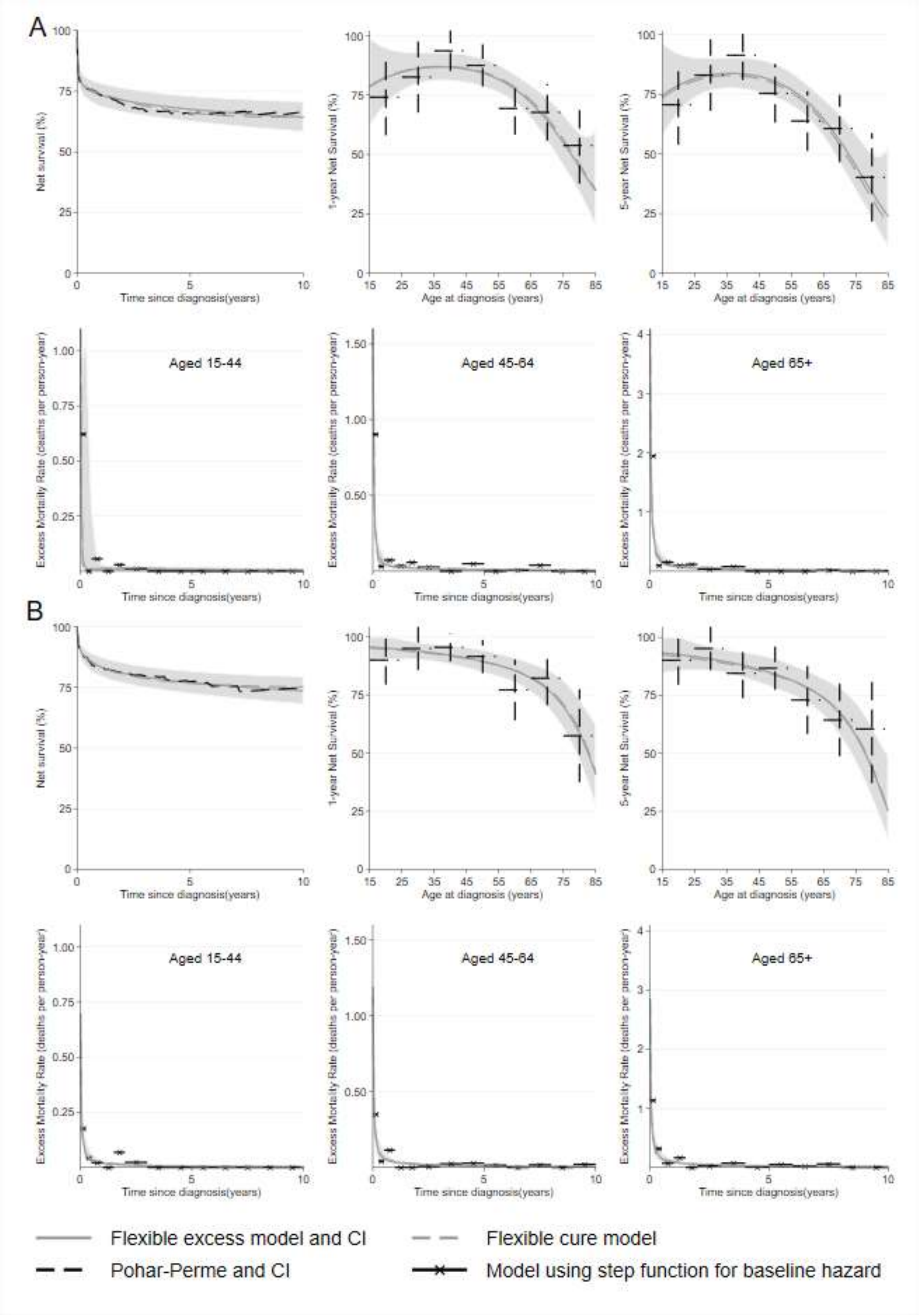

Supplementary Figure 2 - Adequacy of flexible excess model and flexible cure model in acute myeloid leukemia with recurrent cytogenetic abnormalities (AML-RCA): A) Men and B) Women. Net survival (%) over time since diagnosis for all age (Top left graph). Net survival (and CI95%) over age at diagnosis in years at 1-year (Top middle graph) and 5-year (Top right graph). Excess mortality rate in deaths per person-year over time since diagnosis in years in [15-44] age-group (Bottom left graph), in [45-64] age-group (Bottom middle graph) and in 65 years old and more (Bottom right graph).

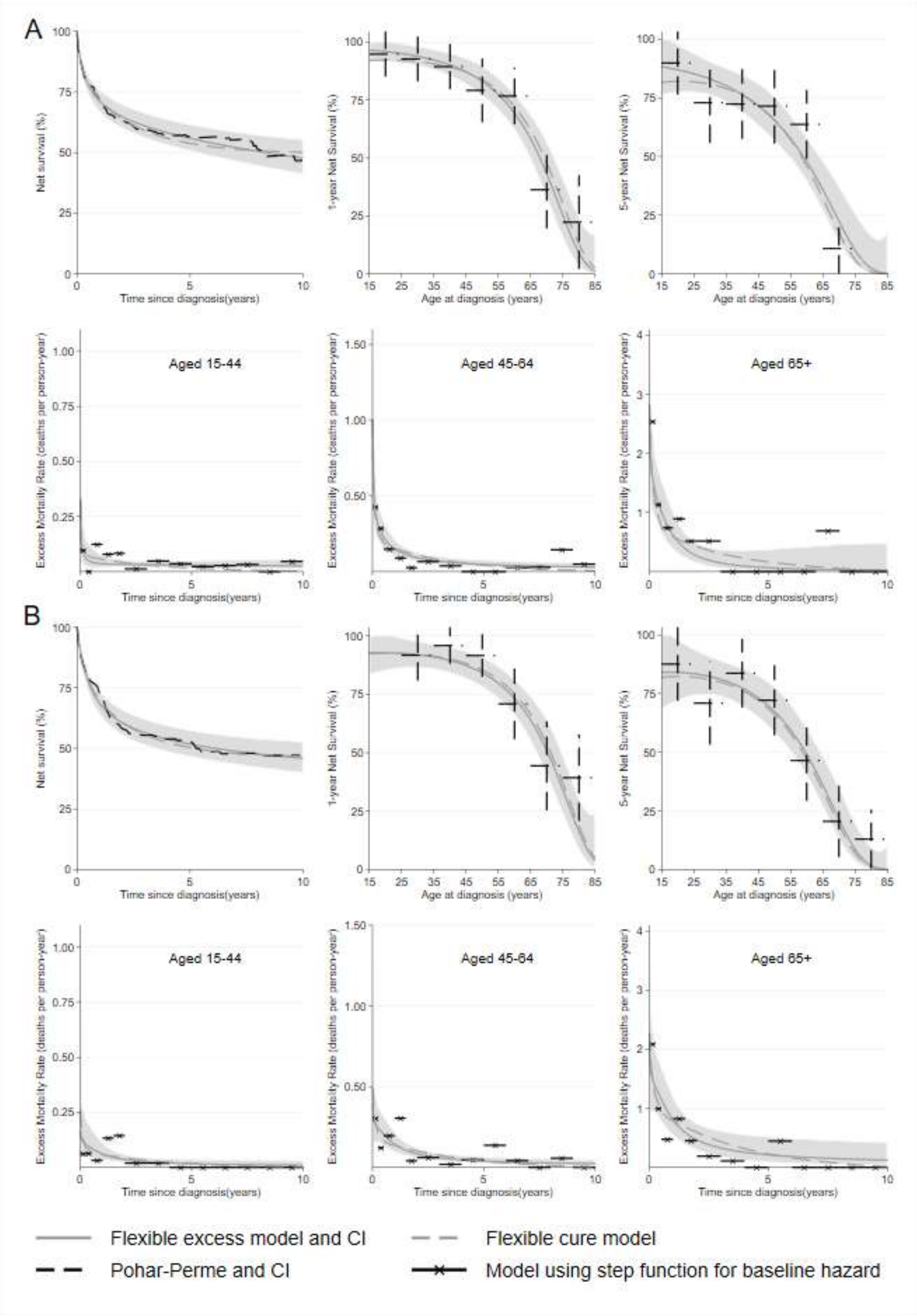

Supplementary Figure 3 - Adequacy of flexible excess model and flexible cure model in acute myeloid leukemia with myelodysplasia-related changes (AML-MRC): A) Men and B) Women. Net survival (%) over time since diagnosis for all age (Top left graph). Net survival (and CI95%) over age at diagnosis in years at 1-year (Top middle graph) and 5-year (Top right graph). Excess mortality rate in deaths per person-year over time since diagnosis in years in [15-44] age-group (Bottom left graph), in [45-64] age-group (Bottom middle graph) and in 65 years old and more (Bottom right graph).

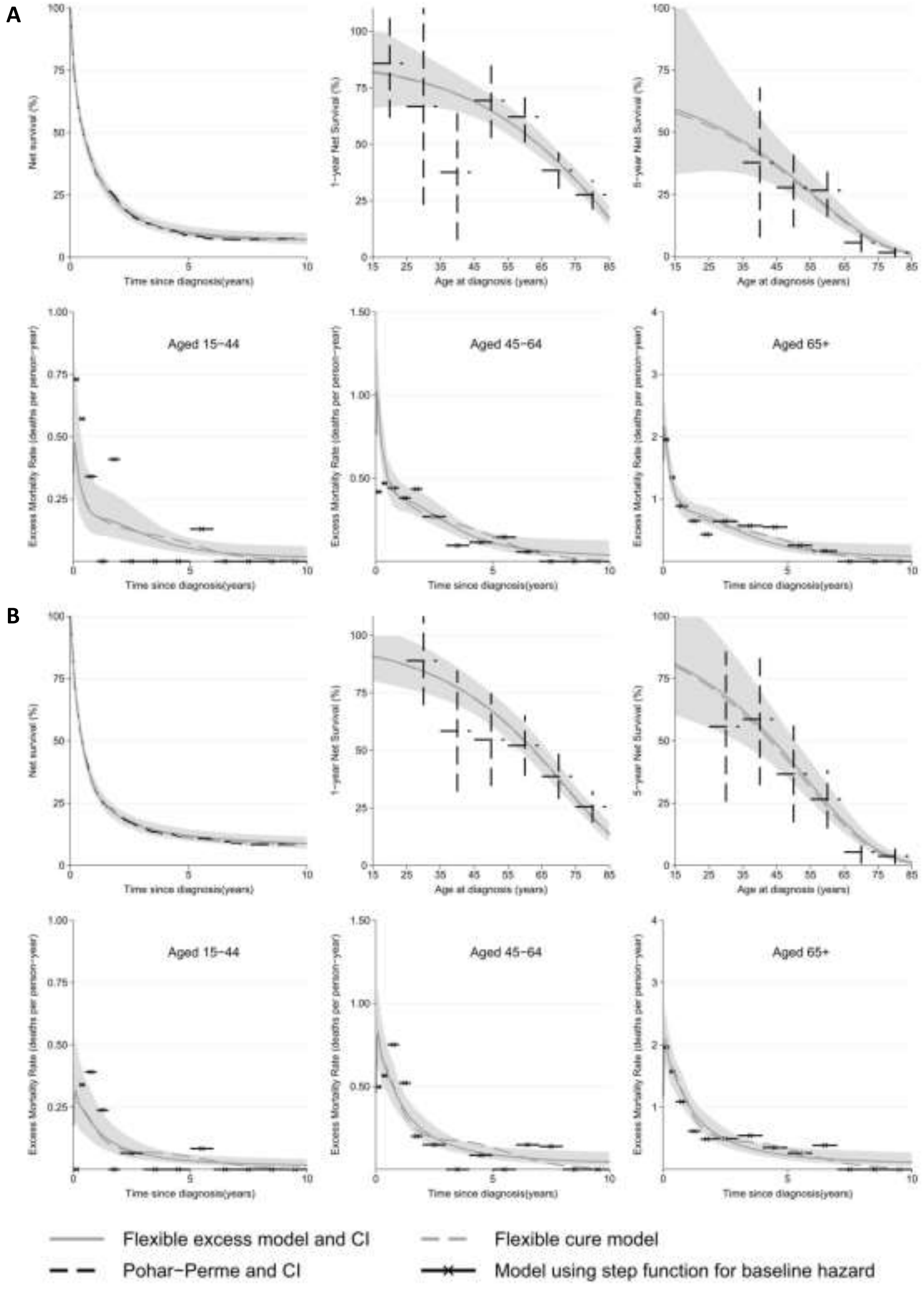

Supplementary Figure 4 - Adequacy of flexible excess model and flexible cure model in therapy-related acute myeloid leukemia, NOS (t-AML): A) Men and B) Women. Net survival (%) over time since diagnosis for all age (Top left graph). Net survival (and CI95%) over age at diagnosis in years at 1-year (Top middle graph) and 5-year (Top right graph). Excess mortality rate in deaths per person-year over time since diagnosis in years in [15-44] age-group (Bottom left graph), in [45-64] age-group (Bottom middle graph) and in 65 years old and more (Bottom right graph).

A

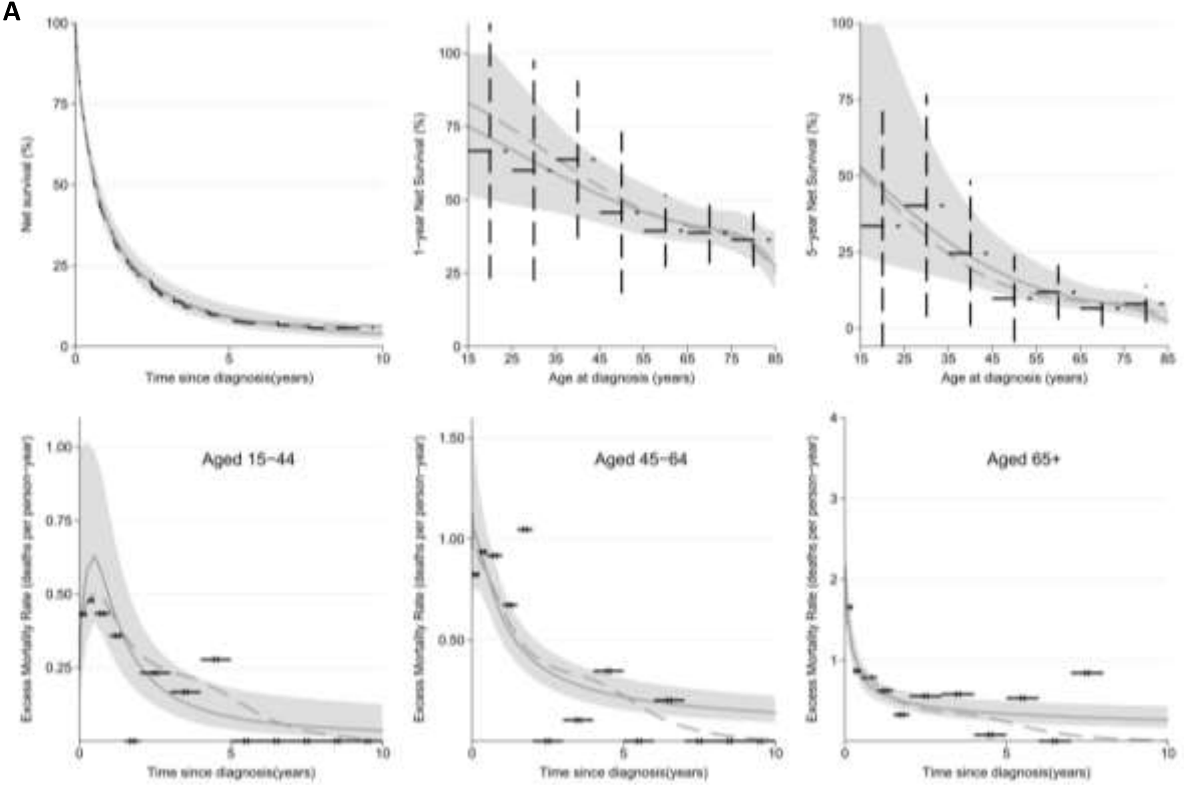

B

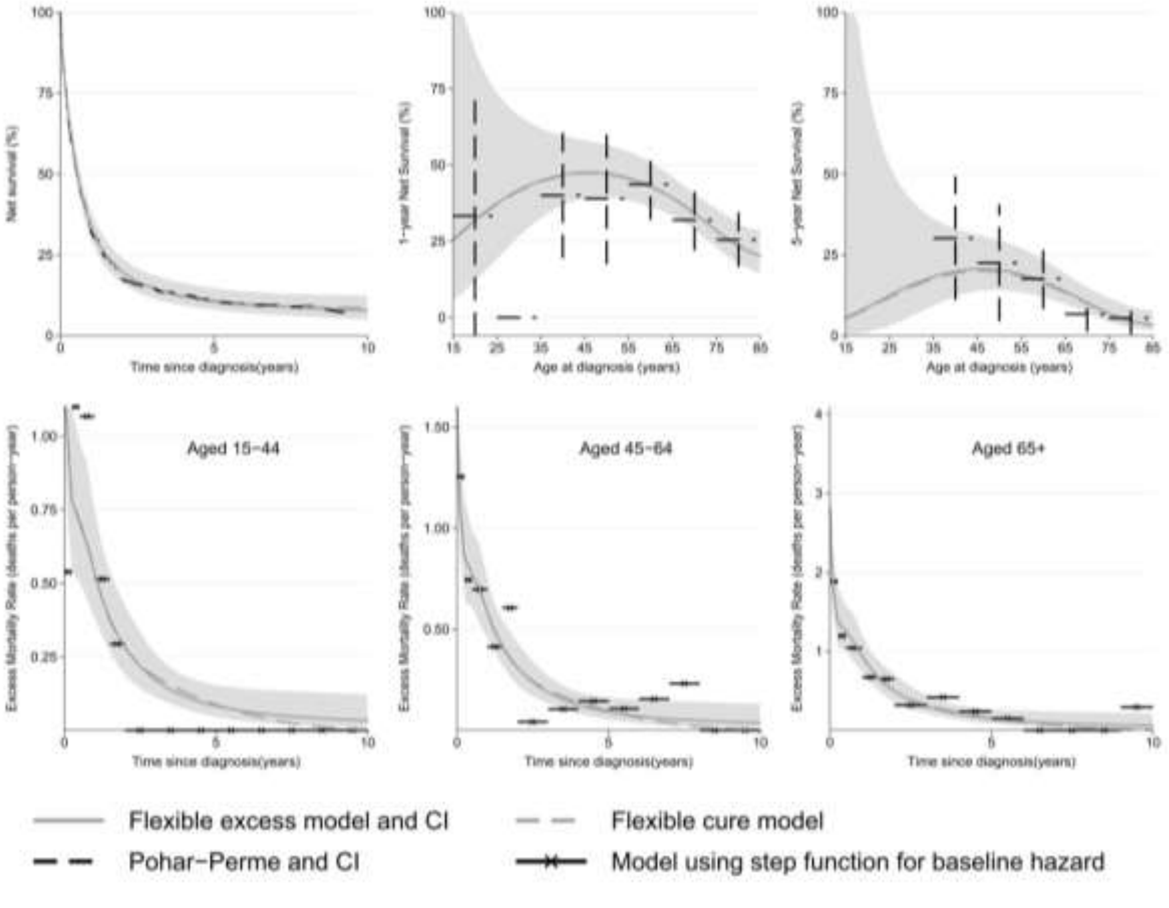

Supplementary Figure 5 - Adequacy of flexible excess model and flexible cure model in Pure erythroid leukemia (AML-M6) in men. Net survival (%) over time since diagnosis for all age (Top left graph). Net survival (and CI95%) over age at diagnosis in years at 1-year (Top middle graph) and 5-year (Top right graph). Excess mortality rate in deaths per person-year over time since diagnosis in years in [15-44] age-group (Bottom left graph), in [45-64] age-group (Bottom middle graph) and in 65 years old and more (Bottom right graph).

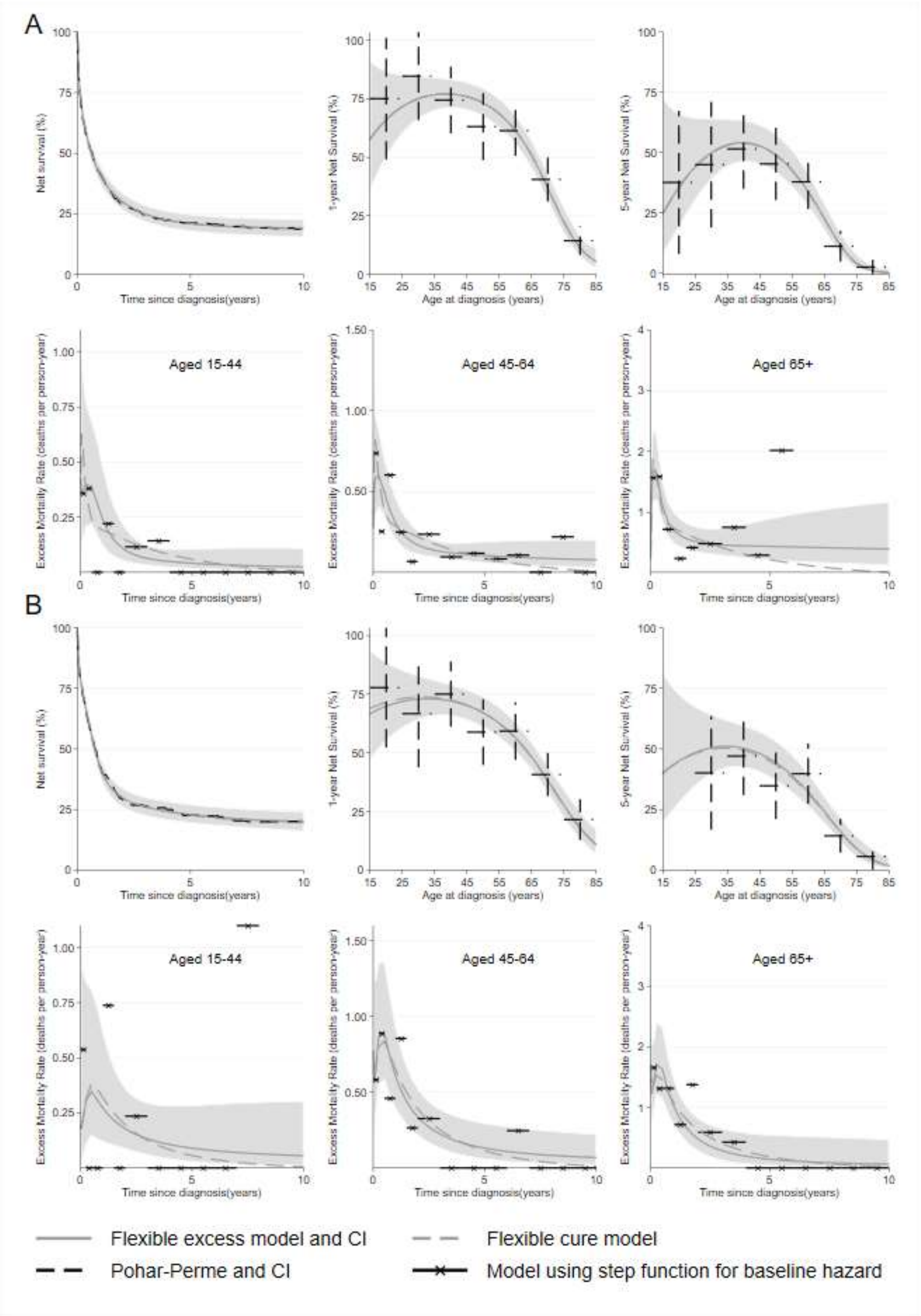

Supplementary Figure 6 - Adequacy of flexible excess model and flexible cure model in Acute Myelomonocytic Leukemia (AML-M4): A) Men and B) Women. Net survival (%) over time since diagnosis for all age (Top left graph). Net survival (and CI95%) over age at diagnosis in years at 1-year (Top middle graph) and 5-year (Top right graph). Excess mortality rate in deaths per person-year over time since diagnosis in years in [15-44] age-group (Bottom left graph), in [45-64] age-group (Bottom middle graph) and in 65 years old and more (Bottom right graph).

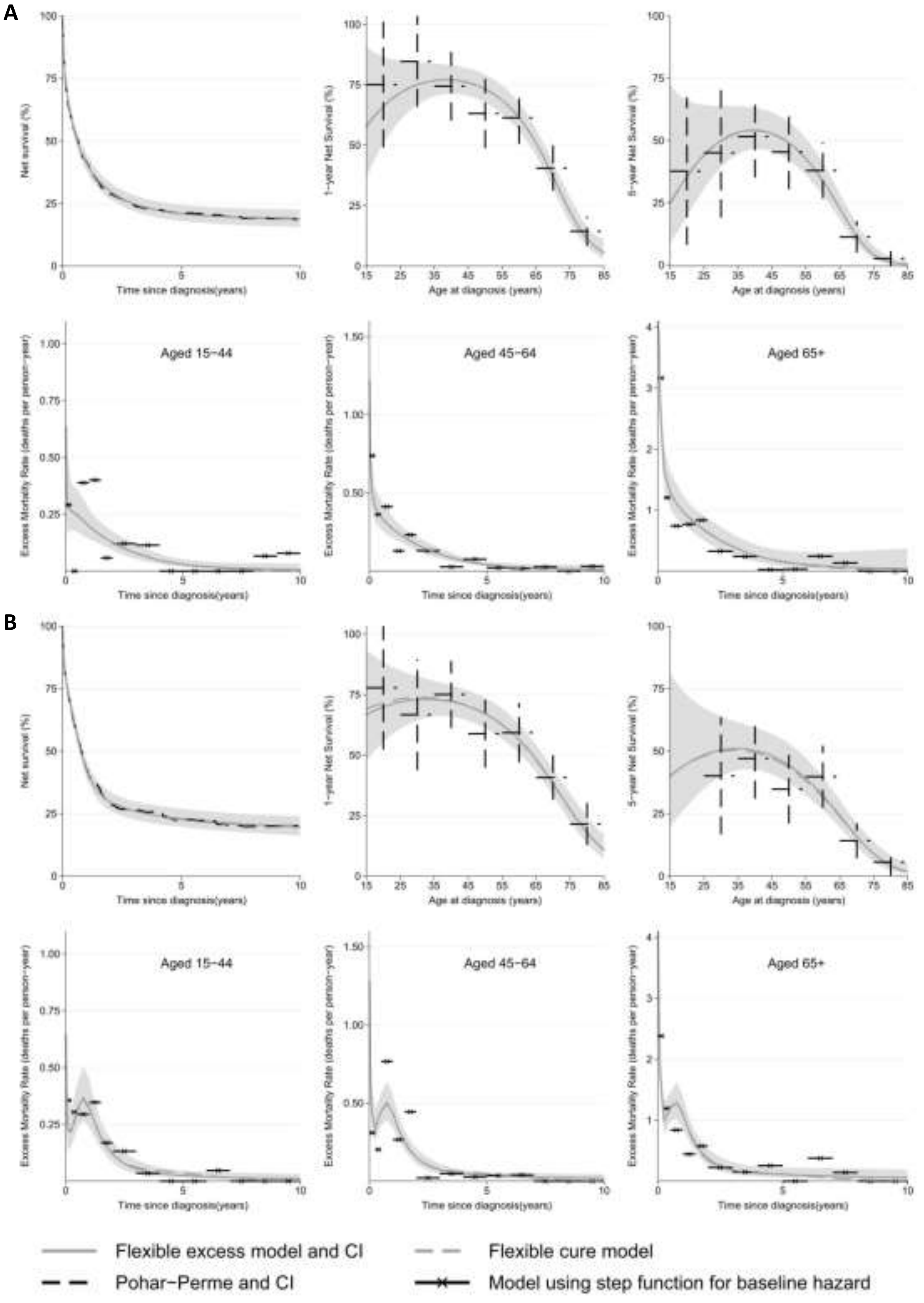

Supplementary Figure 7 - Adequacy of flexible excess model and flexible cure model in acute myeloid leukemia with minimal differentiation (AML-M0): A) Men and B) Women. Net survival (%) over time since diagnosis for all age (Top left graph). Net survival (and CI95%) over age at diagnosis in years at 1-year (Top middle graph) and 5-year (Top right graph). Excess mortality rate in deaths per person-year over time since diagnosis in years in [15-44] age-group (Bottom left graph), in [45-64] age-group (Bottom middle graph) and in 65 years old and more (Bottom right graph).

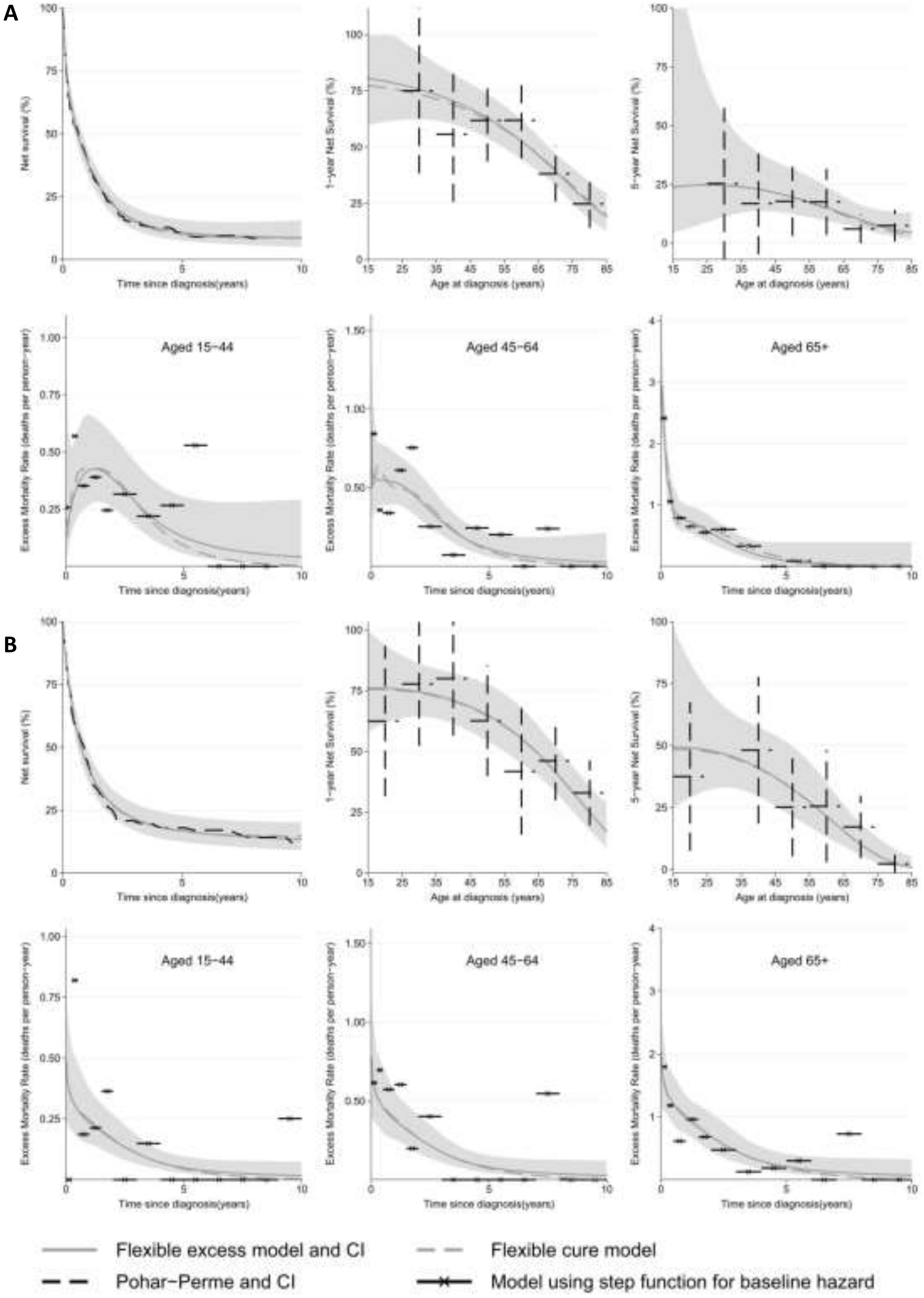

Supplementary Figure 8 - Adequacy of flexible excess model and flexible cure model in acute myeloid leukemia without maturation (AML-M1): A) Men and B) Women. Net survival (%) over time since diagnosis for all age (Top left graph). Net survival (and CI95%) over age at diagnosis in years at 1-year (Top middle graph) and 5-year (Top right graph). Excess mortality rate in deaths per person-year over time since diagnosis in years in [15-44] age-group (Bottom left graph), in [45-64] age-group (Bottom middle graph) and in 65 years old and more (Bottom right graph).

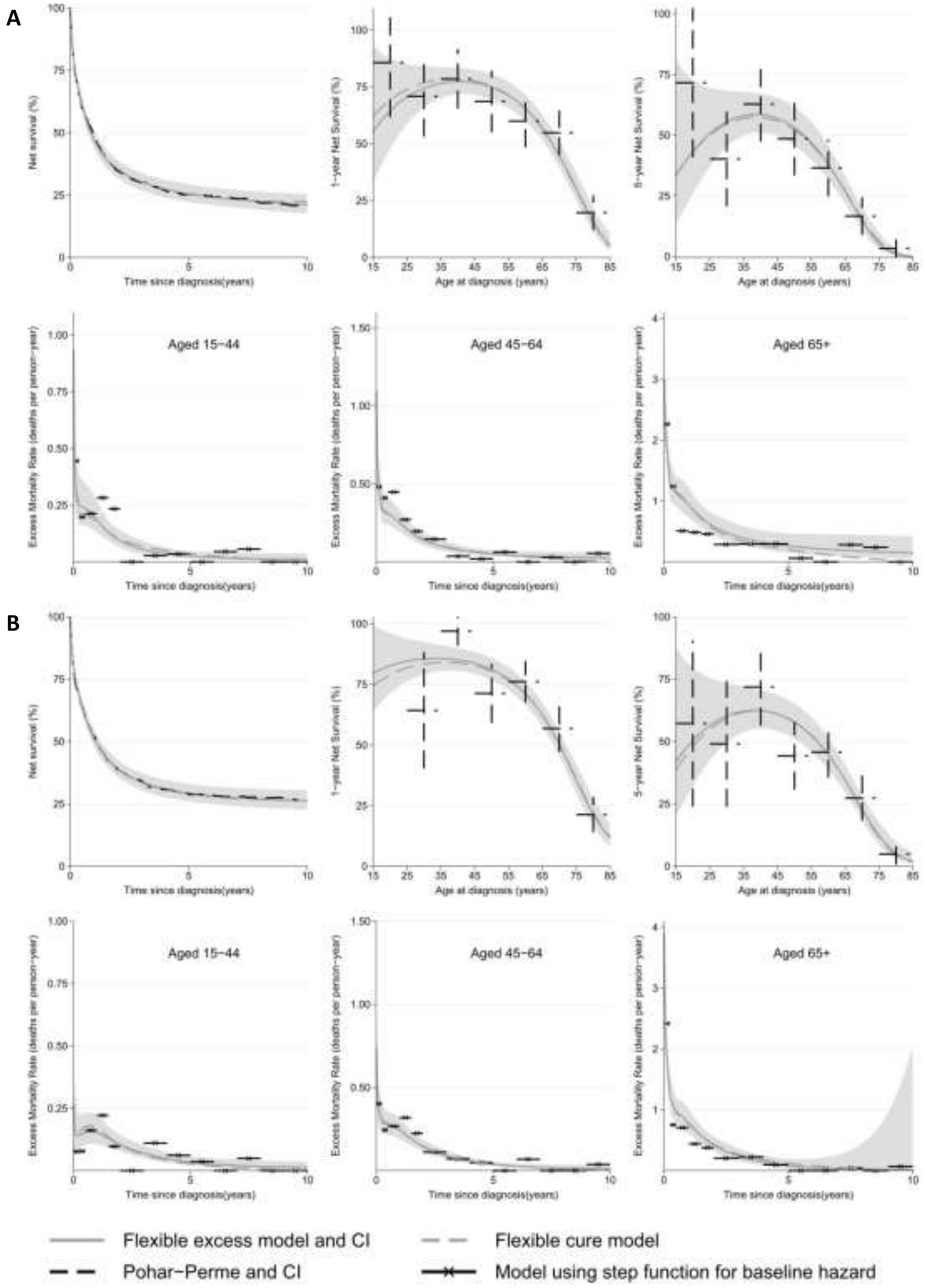

Supplementary Figure 9 - Adequacy of flexible excess model and flexible cure model in acute myeloid leukemia with maturation (AML-M2): A) Men and B) Women. Net survival (%) over time since diagnosis for all age (Top left graph). Net survival (and CI95%) over age at diagnosis in years at 1-year (Top middle graph) and 5-year (Top right graph). Excess mortality rate in deaths per person-year over time since diagnosis in years in [15-44] age-group (Bottom left graph), in [45-64] age-group (Bottom middle graph) and in 65 years old and more (Bottom right graph).

A

B

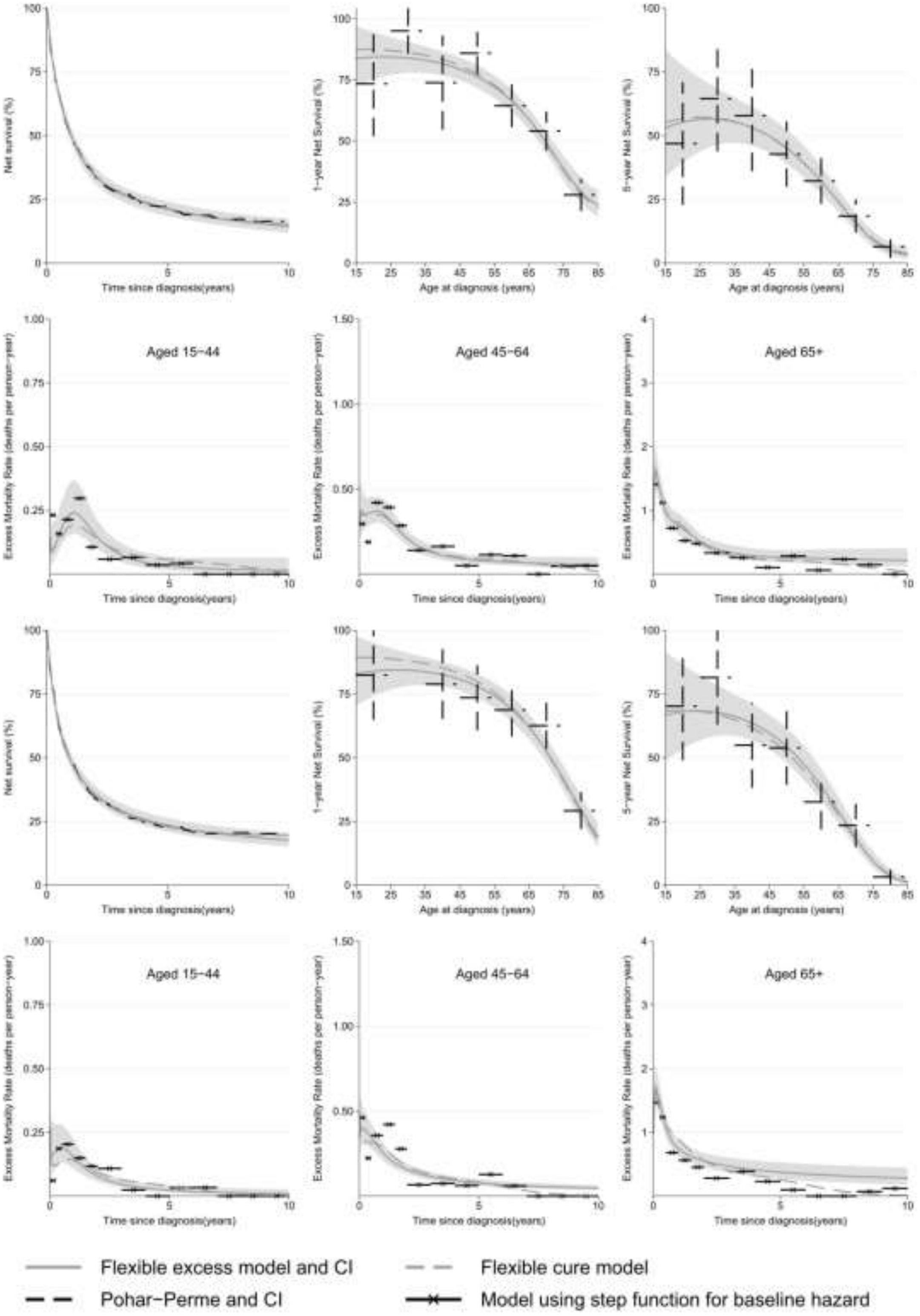

Supplementary Figure 10 - Adequacy of flexible excess model and flexible cure model in acute monoblastic and monocytic leukemia (AML-M5): A) Men and B) Women. Net survival (%) over time since diagnosis for all age (Top left graph). Net survival (and CI95%) over age at diagnosis in years at 1-year (Top middle graph) and 5-year (Top right graph). Excess mortality rate in deaths per person-year over time since diagnosis in years in [15-44] age-group (Bottom left graph), in [45-64] age-group (Bottom middle graph) and in 65 years old and more (Bottom right graph).

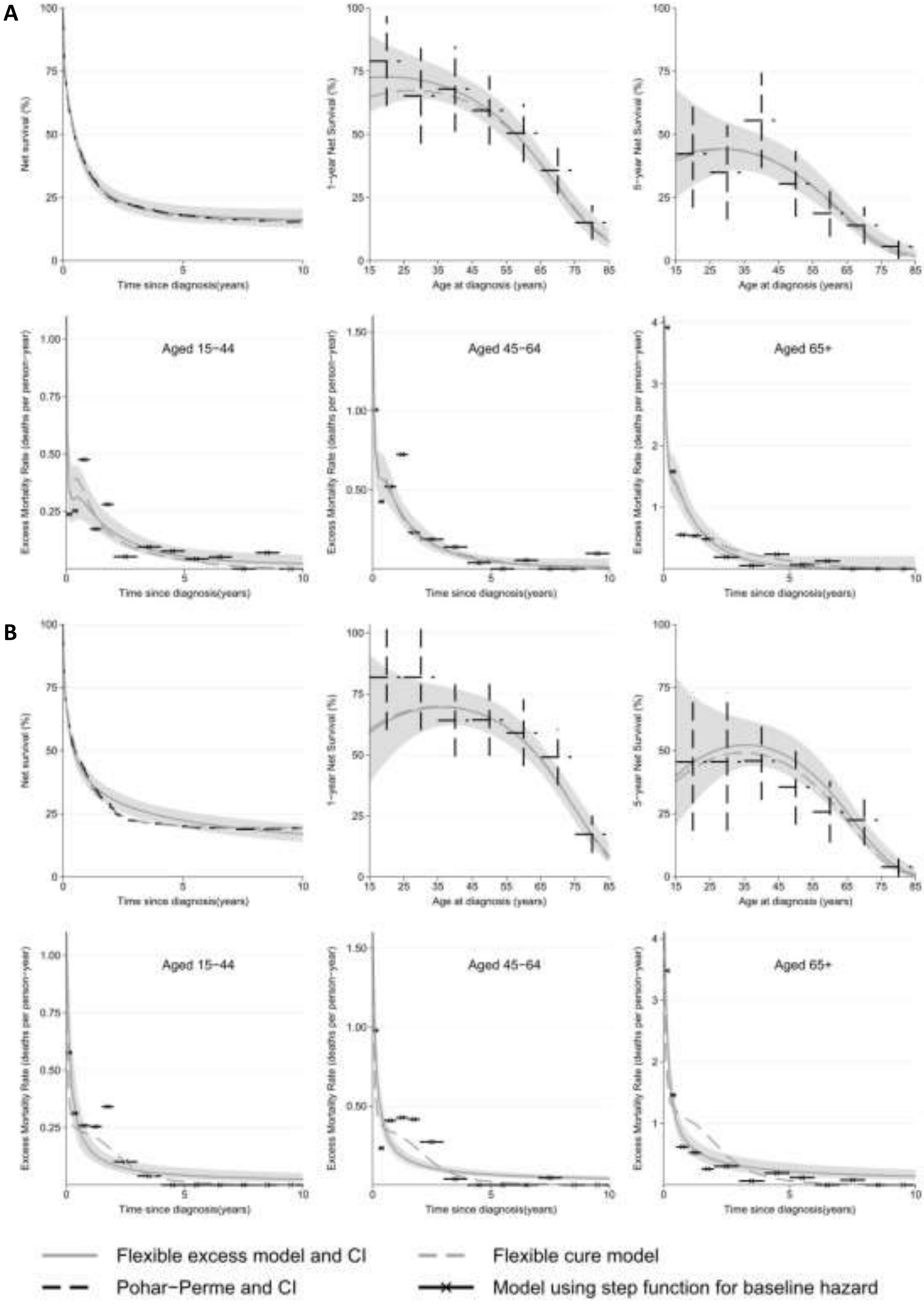

Supplementary Figure 11 - Adequacy of flexible excess model and flexible cure model in Acute Myeloid Leukemia, not otherwise specified (AML-NOS): A) Men and B) Women. Net survival (%) over time since diagnosis for all age (Top left graph). Net survival (and CI95%) over age at diagnosis in years at 1-year (Top middle graph) and 5-year (Top right graph). Excess mortality rate in deaths per person-year over time since diagnosis in years in [15-44] age-group (Bottom left graph), in [45-64] age-group (Bottom middle graph) and in 65 years old and more (Bottom right graph).

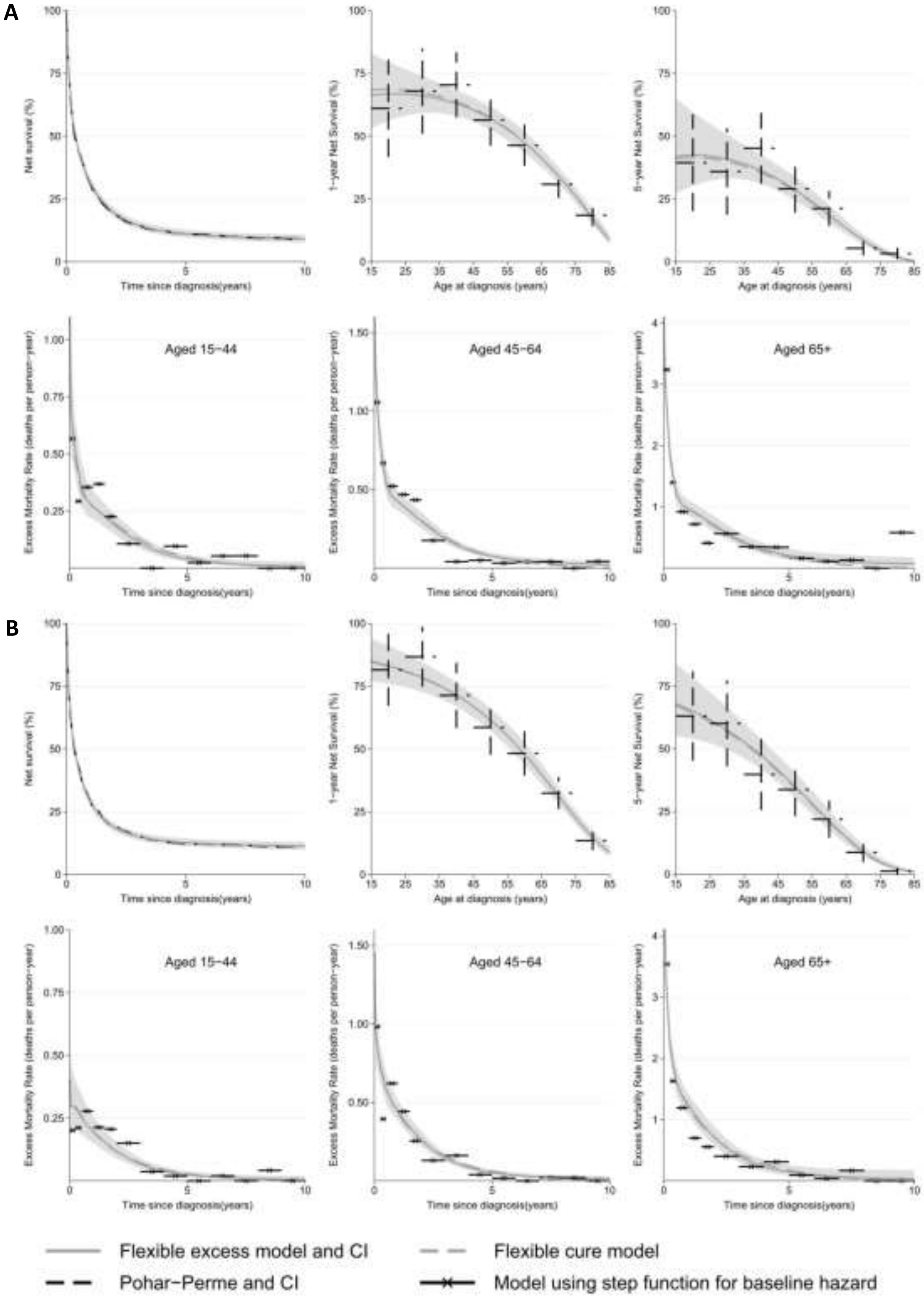

Supplement: Supplementary file 1 [file jcm-10-01657-s001.pdf]
